# The Promise of Systems Biology Approaches for Revealing Host Pathogen Interactions in Malaria

**DOI:** 10.3389/fmicb.2017.02183

**Published:** 2017-11-16

**Authors:** Meghan Zuck, Laura S. Austin, Samuel A. Danziger, John D. Aitchison, Alexis Kaushansky

**Affiliations:** ^1^Center for Infectious Disease Research, formerly Seattle Biomedical Research Institute, Seattle, WA, United States; ^2^Institute for Systems Biology, Seattle, WA, United States; ^3^Department of Global Health, University of Washington, Seattle, WA, United States

**Keywords:** malaria, plasmodium, liver, systems biology, computational modeling, omics-technologies

## Abstract

Despite global eradication efforts over the past century, malaria remains a devastating public health burden, causing almost half a million deaths annually (WHO, 2016). A detailed understanding of the mechanisms that control malaria infection has been hindered by technical challenges of studying a complex parasite life cycle in multiple hosts. While many interventions targeting the parasite have been implemented, the complex biology of *Plasmodium* poses a major challenge, and must be addressed to enable eradication. New approaches for elucidating key host-parasite interactions, and predicting how the parasite will respond in a variety of biological settings, could dramatically enhance the efficacy and longevity of intervention strategies. The field of systems biology has developed methodologies and principles that are well poised to meet these challenges. In this review, we focus our attention on the Liver Stage of the *Plasmodium* lifecycle and issue a “call to arms” for using systems biology approaches to forge a new era in malaria research. These approaches will reveal insights into the complex interplay between host and pathogen, and could ultimately lead to novel intervention strategies that contribute to malaria eradication.

## Introduction

Parasitic diseases infect over half a billion people worldwide, and are a tremendous public health burden. Malaria is the most lethal, causing infection and death primarily in young children in sub Saharan Africa (WHO, 2016). In humans, five *Plasmodium* species are known to cause disease, with the greatest burden arising from infection with *P. falciparum* and *P. vivax*. Despite multifaceted control efforts, the adaptive nature of the *Plasmodium* parasite has confounded vaccine development (Neafsey et al., 2015; Schats et al., 2015), and has contributed to the emergence of widespread drug resistance (reviewed in Blasco et al., 2017).

The life cycle of *Plasmodium* is complex. The parasite cycles between mosquito and mammalian hosts, with elaborate developmental and differentiation processes within each. Every transition represents an opportunity to arrest the parasite, and to stop subsequent life cycle progression. A systematic approach that identifies key components required by the parasite at each stage of its life cycle could ultimately elucidate fundamental pathogenesis strategies, which will aid the development of cohesive intervention approaches. By contrast, any approach that reduces the biology of the parasite to a single antigen or drug target leaves open the possibility of parasite adaptation and, ultimately, intervention failure. Here, we propose a systems biology approach to interrogate the *Plasmodium* parasite that, although not without its challenges, will result in a global view of the host-parasite interactions during key transition states in the life cycle. This view could inform interventions that are not easily circumvented by the parasite and therefore contribute to malaria eradication.

## *Plasmodium* parasites have a complex life cycle that engages multiple host environments

*Plasmodium* infection of mammals begins with injection of the sporozoite into the skin of the vertebrate host during the bite of a female *Anopheles* mosquito. After migration through the skin and entrance into a capillary, sporozoites travel through the blood stream to the liver. The parasite then traverses through the sinusoidal barrier to gain access to hepatocytes (Mota et al., 2001; Ishino et al., 2004; Tavares et al., 2013; Cha et al., 2016; Yang et al., 2017). Once within the liver parenchyma, sporozoites infect a host hepatocyte within which they will reside for the next 2–10 days (reviewed in Kaushansky and Kappe, 2015b; Vaughan and Kappe, 2017). Following liver stage development, parasites exit the liver, re-enter the blood stream and infect erythrocytes. During asexual blood stage infection, parasites undergo cycles of replication, followed by destruction of the host cell. It is this cycle that causes disease symptoms.

**Figure 1:**
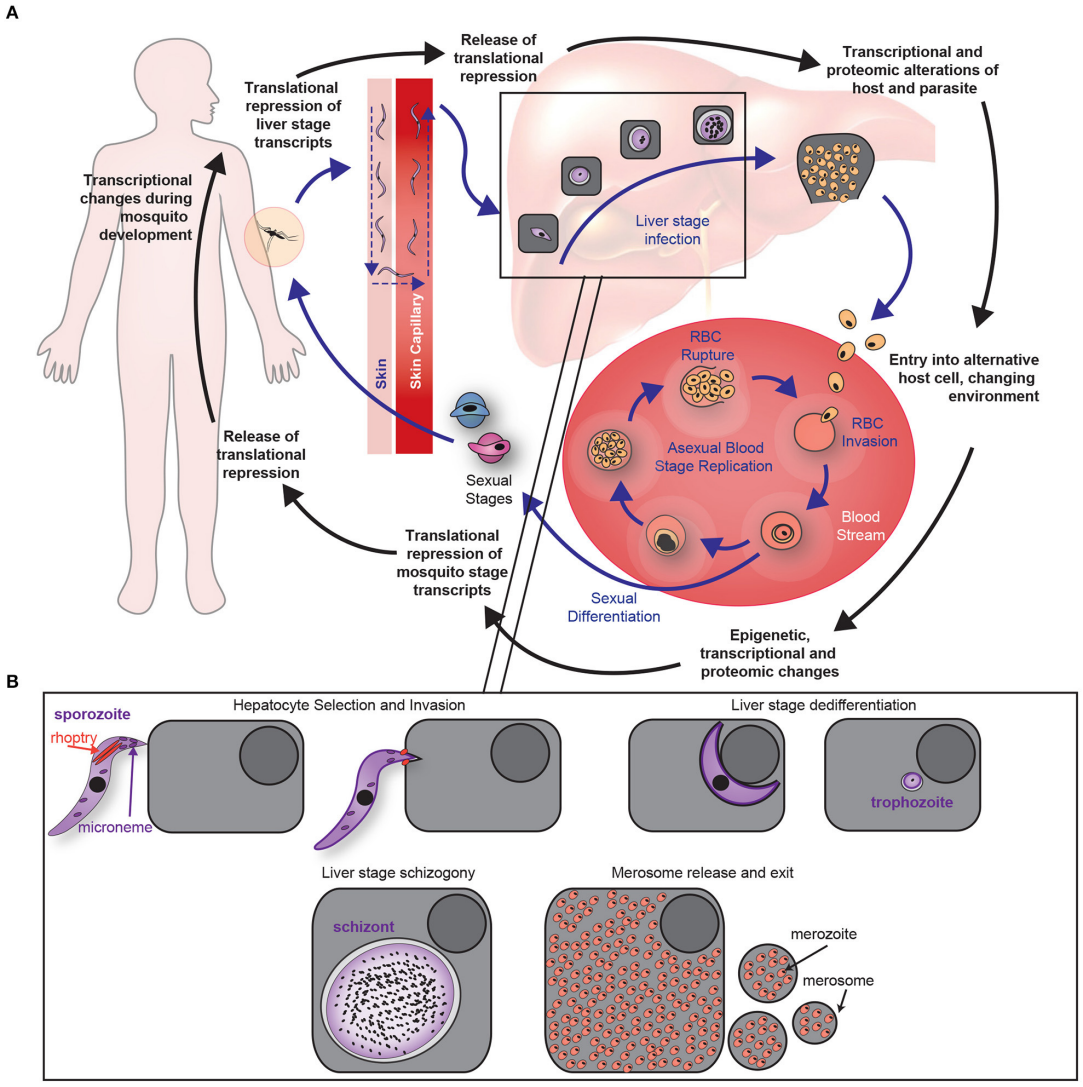
*Plasmodium* life cycle. **(A)** Each stage of the malaria life cycle is accompanied by unique transcriptional or translational changes, which ultimately allow for successful transition to each stage of the life cycle. Red Blood Cell is abbreviated “RBC.” **(B)** Liver stage infection of a hepatocyte is a unique microenvironment that allows the parasite to invad and differentiate into several forms to ensure growth, replication, and eventual egress from the hepatocyte. These key transitions occur in specific subcellular locations during liver stage infection.

During the blood stage, a portion of parasites commit to sexual development (Coleman et al., 2014; Kafsack et al., 2014; Sinha et al., 2014; Poran et al., 2017) and initiate a differentiation process that occurs largely in the bone marrow (Joice et al., 2014). Once female and male forms have nearly completed maturation, they re-enter the blood stream and are transmitted to mosquitoes. In the mosquito midgut, fertilization occurs, generating a motile diploid (ookinete), which then replicates its DNA and develops into a stationary oocyst. Sporozoites then form within the midgut oocyst, become motile, and travel to the salivary glands. Once within the salivary glands, the parasite is transmitted to the next mammalian host during a blood meal. Each of these stage transitions is initiated by, and induces, broad, systematic changes that alter cellular behaviors (Table 1, Figure 1). Yet, these changes cannot be fully represented by any single transcript or individual cellular measurement. Rather, comprehensive changes within interconnected networks occur on multiple scales. This includes changes in gene regulatory networks, protein interactions with other biomolecules, and morphological variation of host and parasite subcellular structures. Together, these changes drive stage transitions. The goal must therefore be to establish a comprehensive picture of the host and parasite effector molecules and networks that are required to facilitate life cycle transitions.

**Table 1:** Stage transitions in the *Plasmodium* life cycle.

**Life cycle stage transition**	**System-level alteration reported**	**References**
Development from midgut sporozoite to salivary gland sporozoite	Transcriptome changes	Matuschewski et al., 2002; Mikolajczak et al., 2008
Transmission between mosquito and mammalian host	Translational repression	Zhang et al., 2010; Gomes-Santos et al., 2011; Muller et al., 2011; Lindner et al., 2013; Silvie et al., 2014; Silva et al., 2016
Development through Liver Stage	Transcriptome and proteome changes	Tarun et al., 2008; Albuquerque et al., 2009; Vaughan et al., 2009
Exit from Liver Stage and Entry into Blood Stage	Transcriptome changes	Tarun et al., 2008
Differentiation into sexual forms	Epigenetic and Transcriptome changes	Coleman et al., 2014; Kafsack et al., 2014; Sinha et al., 2014; Poran et al., 2017
Transmission from mammalian to mosquito host	Translational repression	Mair et al., 2006; Guerreiro et al., 2014; Lasonder et al., 2016
Gametocyte to gamete transformation	Proteome changes	Khan et al., 2005

## *Plasmodium* parasites significantly alter the biology of their hosts

To illustrate the need to comprehensively evaluate changes during the *Plasmodium* life cycle, we will consider one stage of the complex life cycle of the parasite in detail—the Liver Stage of infection. Once within the liver sinusoid, the parasite traverses through phagocytic Kupffer cells, liver-resident macrophages, and liver endothelial sinusoidal cells, to access hepatocytes, while avoiding phagocytosis (Mota et al., 2001; Ishino et al., 2004; Usynin et al., 2007; Tavares et al., 2013; Cha et al., 2016; Yang et al., 2017). Once in the liver parenchyma, the parasite continues to traverse through several hepatocytes before selecting a suitable host for invasion. While the precise properties that make one hepatocyte more hospitable than another remain unknown, altered levels of specific hepatocyte receptors dramatically alter infection rates (Silvie et al., 2003; Ishino et al., 2004; Rodrigues et al., 2008; Yalaoui et al., 2008a; Kaushansky et al., 2015).

Following establishment of an intracellular niche within the hepatocyte, *Plasmodium* replicates extensively, stretching the hepatocyte to 50–100 times its normal volume (Shortt and Garnham, 1948; Vaughan et al., 2012). This rapid expansion is surprising, given the cell's strict cell size regulations under normal conditions (Sinturel et al., 2017). This observation suggests that *Plasmodium* effectively overwrites the hepatocyte's hardwiring to exert massive influence over the host cell. *Plasmodium* likely disrupts a multitude of classical signaling pathways during infection, only a small fraction of which have been described (Kaushansky et al., 2013a,b; Ruivo et al., 2016). Interrogating single proteins in a pathway to determine functionality is limiting in this context, and ignores secondary effects within the complex cell system. Instead, it is critical to comprehensively and quantitatively evaluate changes that occur during infection to illuminate mechanisms of control employed by the parasite.

## What can systems biology do for malaria?

**Figure 2:**
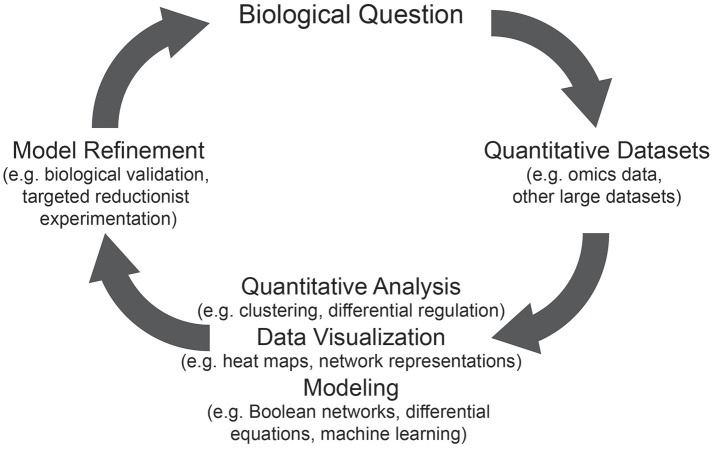
The processes involved in generating systems biology-informed models. To inform the biological question, quantitative datasets are generated, which are then used in quantitative analysis, data visualization, and modeling to describe how the system behaves. These tools can be used interchangeably and/or in succession before further refining the model. Refinement of the model can then provide new biological insights.

Understanding biology is a systems-level problem. Interactions between components of a system lead to the emergence of properties that cannot be understood from the study of the components individually. The study of systems biology is predicated on two basic assumptions. First, that the whole is far greater than the sum of its parts; and second, that a more comprehensive understanding of the components and their relationships within a system will allow for more accurate predictions of the system's behavior. It is through this lens that systems biology aims to determine the relationships and interactions of the components of a system. In practice, systems biology is a set of principles and processes by which we take complex systems apart and put them back together, with the aim of understanding the properties of the entire biological system. The approach generally starts with the systematic and comprehensive identification and quantification of molecules, called omics datasets, as a biological system transitions from one state to another. Initially, these data are evaluated using standard statistical tools, resulting in ordered lists, with significance values for each observed difference. These data provide the basis for the deployment of simple tools such as pathway analysis and clustering to interpret the data, or more complex analysis, such as regression or inference methods, to suggest causal or correlative relationships between components. These approaches can, and have, identified major molecular players at each stage, but fall short of a detailed and comprehensive understanding. Visualizing the data is also important for generating insights and predictive models that describe key determinants of the stage transition being interrogated (Figure 2). Predictions that are generated are then tested, often using “classic” or “reductionist” approaches. This process results in the refinement of both the model, and of our biological understanding. Molecular details are important, and systems biology must not ignore them. Examining the individual components of a system allows us to understand their molecular and physicochemical properties, as well as the function of the components in context of the entire system (Van Regenmortel, 2004). As the data that informs a model becomes more detailed, the predictions generated become more mechanistic. This level of insight is critical for rational intervention.

Modeling is not unique to systems biology. Indeed, all scientists generate “models,” sometimes in the form of cartoons to aid in the design of the next line of inquiry. Systems biology models are often in the form of networks composed of balls and sticks, where balls (also called nodes) represent genes or proteins, and the sticks (or lines, also called edges) between them represent a relationship between molecular players. These simple visualizations can themselves facilitate the development of novel hypotheses. Many representations allow scientists to superimpose multiple types of data onto these networks (for example, molecule types, confidence of the interactions or subcellular organization). There are many popular and facile tools for these network visualizations (reviewed in Gehlenborg et al., 2010; Pavlopoulos et al., 2017). Once established, these networks can be mined to design subsequent experiments, and also used as a foundation for more complex models of the dynamics of molecular interactions and information flow. Depending on the complexity and the question to be addressed, models can take many forms. Some widely used approaches include Boolean networks, ordinary differential equations, and stochastic simulations. Different model classes involve different approximations, assumptions and levels of granularity. All models are best informed by quantitative, high quality, and biochemical data. While omics approaches can contribute to a more comprehensive view than possible with classical biochemical approaches, many models also incorporate data from rigorous reductionist approaches. Regardless of model class, the power of modeling lies in its capacity to capture insights that are difficult to reach through intuition alone.

Many of the technological and computational tools of systems biology are modular, and the resulting data can be integrated in different ways to inform the biological question (Danziger et al., 2014). Indeed, modeling biological data increasingly aims to incorporate a range of types of information, which monitor changes at different scales. This allows the researcher to determine what types of data are most informative when predicting a biological outcome of interest (Hwang et al., 2005a,b; Janes et al., 2005; Bonneau et al., 2007; AlQuraishi et al., 2014) and design subsequent experiments accordingly. Nevertheless, in most applications, applying computational analysis to quantitative datasets enables predictions (or new hypotheses) about how a perturbation, such as a gene deletion, drug treatment, or new environment, will influence the system as a whole.

### The role of quantitative and comprehensive datasets in malaria research

In the case of malaria, numerous studies have generated omics data during life cycle transitions (Table 1). These include the cataloging of genes (genomics), mRNA transcripts (transcriptomics), translated protein (proteomics), metabolites (metabolomics), and translational repression/de-repression of transcripts as the parasite transitions through its life cycle stages. The genome of the *Plasmodium* parasite was initially published in 2002 (Gardner et al., 2002), and has been refined since. Initial transcriptomes and proteomes of *P. yoelii* and *P. berghei* Liver Stages have been generated, which have provided lists of the components involved in liver stage development, and further revealed the requirement of fatty acid synthesis from both the parasite and host during liver infection (Tarun et al., 2008; Albuquerque et al., 2009; Vaughan et al., 2009).

Insights that originate from transcriptomic analysis of Liver Stage infection reveal that Type I interferons and ER stress are systematically upregulated during liver stage infection and can modulate the level of liver stage infection (Liehl et al., 2014; Miller et al., 2014; Inacio et al., 2015; Kaushansky and Kappe, 2015a). Additional information can be obtained by monitoring changes in the parasite and host during infection under different environmental conditions. These, and related datasets can inform models that predict causality and cellular outcomes. The goal of this effort would be to identify networks of parasite and/or host factors that facilitate the development or demise of the parasite during its infection of the liver.

### Protein-protein interactions

A major goal of host-pathogen studies is to elucidate specific interactions that dictate success or failure of the pathogen. While transcriptomics and proteomics can catalog changes that occur in the infected cell, a list of alterations alone does not provide mechanistic insight, and is unsatisfying to most cell biologists. Databases of protein interactions in many organisms, including humans, are becoming highly populated (Hein et al., 2015; Huttlin et al., 2015). Yet, many immunopurification—mass-spectrometry (IP-MS) based approaches to study protein-protein interactions do not meet the standard of quantitative and comprehensive. As datasets become larger, statistical tools can be used to predict which interactions are more likely to be specific, compared to commonly identified (abundant or promiscuous) proteins (Mellacheruvu et al., 2013). The pitfalls of qualitative and low throughput data have been partially overcome in model organisms such as yeast, where whole-genome GFP tag libraries have been generated and used in IP-MS experiments, although even these datasets remain incomplete, and are error prone (Ghaemmaghami et al., 2003; Huh et al., 2003; Mellacheruvu et al., 2013).

More sophisticated approaches designed to distinguish between bona fide and spurious interactions are being developed and applied. For example, Isotopic Differentiation of Interactions as Random or Targeted (I-DIRT) and variants (Tackett et al., 2005; Selbach and Mann, 2006; Trinkle-Mulcahy et al., 2008; Byrum et al., 2012; Trinkle-Mulcahy, 2012) exploit isotopic labeling and immunopurification to distinguish between interactions that occur before cell lysis, from those interactions that are introduced during the purification process. While these approaches improve confidence in interactions, they are neither widely adopted, nor have they been applied in genome scale studies.

### The role of imaging in defining quantitative stage transitions

Since the initial discovery of liver stage parasites by microscopy in 1948 (Shortt and Garnham, 1948), imaging has been an invaluable tool of malaria research. However, most common imaging methods are neither quantitative nor comprehensive, limiting their capacity to inform modeling approaches. This is particularly troubling for applying a systems biology approach, as cellular outcomes are what we aim to predict, but are often poorly defined. Traditional imaging also falls short of reaching the temporal resolution necessary to elucidate the dynamic cellular processes during invasion and throughout liver stage infection.

A number of new imaging modalities enhance our ability to increase resolution, quantification and throughput. A comprehensive review of the advances made in increasing throughput, quantification and resolution in the imaging field is outside the scope of this review, we will highlight some examples that are particularly relevant to malaria research. One example, correlated light microscopy and electron microscopy (CLEM) combines fluorescence microscopy with electron microscopy, thereby increasing the throughput of monitoring rare events like liver stage infection at EM-level resolution (van Rijnsoever et al., 2008), and has already been applied to monitor liver stage development (Grutzke et al., 2014). Intravital imaging (IVM) has been adapted for malaria research and facilitates analysis of live tissue with microscopic resolution to reveal cellular responses that closely mimic *in vivo* infection, both spatially and temporally (Pittet and Weissleder, 2011; De Niz et al., 2017). Additional instrumentation, such as the Lattice Light Sheet Microscope (Betzig et al., 2006), enhances temporal and spatial resolution, with applications in both *in vivo* and *in vitro* systems, which could enable a more quantitative assessment of cellular outcomes.

### The power of new genetic tools and screens in determining function

An essential component of systems biology is the experimental testing of predictions made by modeling efforts. This testing is greatly assisted by the capacity to perform genetic perturbations. Indeed, one of the major shortfalls of employing systems biology is that testing predictions is largely performed by single candidate-based approaches, and thus often fails to recapitulate the complexity of the system. In many cases, it remains difficult to determine if the model is incorrect, or if reductionist approaches cannot fully capture the emergent properties associated with a complex system. New genome-editing approaches, like CRISPR/Cas, can assess multiple perturbations in combination, in both mammalian and parasite genomes, which will facilitate testing more complex models (Cong et al., 2013; Mali et al., 2013; Ghorbal et al., 2014; Wagner et al., 2014; Lu et al., 2016).

In addition to evaluating individual or groups of gene candidates for function, new genome-editing approaches also have the ability to globally evaluate both host and parasite genes. Whole genome CRISPR/Cas9 knockout screens are now common in mammalian cells (Cong et al., 2013; Mali et al., 2013) and can be adapted to the *Plasmodium* genome. The *Plasmodium* Genetic Modification Project (PlasmoGEM), a new community resource from the Wellcome Trust Sanger Institute, aims to produce new tools for the genetic modification of malaria parasites at genome scale. This resource has already demonstrated that two-thirds of *P. berghei* genes contribute to normal blood stage development (Bushell et al., 2017). Subsequent studies should not only focus on the role of parasite genes in other life cycle stages, but also interrogate the role of host genes during each stage of parasite development.

## Key questions and findings in malaria liver stage biology

The existing literature provides a basis upon which global experiments can be designed and modeled, and also highlights the most critical questions that remain. Given the potential and increasing power of systems biology, the challenge lies in how to use this approach to bolster the rich collection of findings that have been amassed by the *Plasmodium* research community, and address hurdles that have been unattainable by more traditional approaches. In this next section, we focus on some of the key findings on liver stage malaria with an emphasis on questions that remain.

### Hepatocyte invasion

During hepatocyte invasion, the parasite attaches to the host cell, at least partially through circumsporozoite protein (CSP), which interacts directly with highly sulfated proteoglycans (HSPGs) on the cell surface to trigger CSP cleavage, inducing the sporozoite to switch to an invasive state (Table 2A) (Coppi et al., 2007, 2011). Thrombospondin-related anonymous protein (TRAP) is also involved in this process (Kappe et al., 1999; Matuschewski et al., 2002; Morahan et al., 2009). Additionally, *Plasmodium* proteins P36 and P52 play a role in invasion, parasitophorous vacuole membrane (PVM) formation, and protecting the host against apoptosis (Ishino et al., 2005; van Dijk et al., 2005; Ploemen et al., 2012). How each of these factors works in concert to facilitate productive invasion of the hepatocyte remains unknown.

**Table 2:** Determinants of hepatocyte liver stage infection: **(A)** *Plasmodium* determinants of infection and **(B)** Host determinants of infection.

**Host/Parasite factor**	**Stage of infection**	**Main findings**	**References**
**(A)**			
SPECT	Traversal	Essential for cell traversal	Ishino et al., 2004, 2005
PLP1 (SPECT2)		Essential for cell traversal	Ishino et al., 2004, 2005
CelTOS		Hypothesized to play a role in the exit step of traversal	Kariu et al., 2006
TRAP-like protein (TLP)		TLP-deficient sporozoites show a diminished ability to traverse	Moreira et al., 2008
PL (UIS10)	Hepatocyte Invasion	PL-deficient sporozoites show reduction in Liver Stage burden	Bhanot et al., 2005
Circumsporozoite protein (CSP)		Multiple roles in motility and invasion, including transition from traversing state to invasive state	Coppi et al., 2007
P36		Contributes to PVM formation	Ishino et al., 2005; Labaied et al., 2007
P52/P36p		Contributes to PVM formation	Ishino et al., 2005; Labaied et al., 2007
Cysteine proteases		Inhibition of sporozoite cysteine proteases completely inhibits infectivity	Coppi et al., 2005
Calcium Dependent Protein Kinase-6 (CDPK-6)		Sporozoites from CDPK-6-deficient parasites show decrease in invasion and CSP cleavage	Coppi et al., 2007
TRAP		Direct role in invasion through attachment with cytoplasmic tail	Kappe et al., 1999; Matuschewski et al., 2002; Morahan et al., 2009
Upregulated in Sporozoite 4 (UIS4)	Liver stage Development	UIS4-deficient *P. berghei* parasites severely impaired in Liver Stage development	Mueller et al., 2005
Upregulated in Sporozoite (UIS3)		UIS3-deficient parasites severely impaired in Liver Stage development. UIS3 has been hypothesized to play a role in fatty acid uptake	Mikolajczak et al., 2007
EXP1		Interacts with host Apolipoprotein H to promote liver stage development	Sa et al., 2017
LISP2		Hypothesized to be involved in merozoite formation and exported to host cytosol	Orito et al., 2013
B9		P9 mutants show liver stage growth arrest	Annoura et al., 2014
Sequestrin		Mutants lacking sequestrin show a reduction in liver stage development	Annoura et al., 2014
MSP1		Conditional mutagenesis of MSP1 in sporozoites impaired merozoite formation	Combe et al., 2009
LISP1	Hepatocyte Exit	In *P. berghei*, LISP1 is required for lysis of the PVM prior to egress	Ishino et al., 2009
SUB1		SUB1-deficient *P. berghei* parasites fail to rupture the PVM prior to egress	Tawk et al., 2013
**(B)**			
CD68	Traversal	Putative receptor of Kupffer cells, gateway for liver stage infection	Cha et al., 2016
Hepatocyte Growth Factor	Hepatocyte Invasion	Secretion of HGF renders *P. berghei* host hepatocytes susceptible to infection	Carrolo et al., 2003
CD81		Required on hepatocytes for *P. yoelii* invasion with PVM formation	Silvie et al., 2003
Cholesterol		Involved in assembly of CD81 microdomains on the cell surface	Silvie et al., 2003, 2006, 2007
HSPGs		Binds CSP, increased sulfation on HSPGs triggers invasion of migrating sporozoite	Frevert et al., 1993; Coppi et al., 2007
EphA2		Engages parasite protein P36 to facilitate hepatocyte invasion	Kaushansky et al., 2015
Scavenger Receptor B1		Required for CD81 microdomain formation, additional roles independent of CD81 for *P. berghei* and *P. vivax*	Rodrigues et al., 2008; Yalaoui et al., 2008a; Manzoni et al., 2017
HGF/MET signaling	Liver Stage Development	Prevents the apoptosis of *P. berghei* infected cells, promoting successful infection	Leiriao et al., 2005
Endosomes and lysosomes		Endosomes and lysosomes are localized around the PVM during development	Lopes da Silva et al., 2012; Grutzke et al., 2014
Phosphatidylcholine		Required for correct localization of proteins within the PVM; important for parasite survival	Itoe et al., 2014
P53		Decreased levels of P53 are important for successful Liver Stage infection.	Kaushansky et al., 2013b
Apolipoprotein H		Interacts with parasite protein EXP1 to promote successful Liver Stage infection	Sa et al., 2017
ALK4		Knockdown reduces Liver Stage infection	Arang et al., 2017
CAMKK2		Knockdown reduces Liver Stage infection	Arang et al., 2017
CSK		Knockdown reduces Liver Stage infection	Arang et al., 2017
FGFR4		Knockdown reduces Liver Stage infection	Arang et al., 2017
FLT1		Knockdown reduces Liver Stage infection	Arang et al., 2017
FLT3		Knockdown reduces Liver Stage infection	Arang et al., 2017
IKBKB		Knockdown reduces Liver Stage infection	Arang et al., 2017
IRAK1		Knockdown reduces Liver Stage infection	Arang et al., 2017
MAPK1		Knockdown reduces Liver Stage infection	Arang et al., 2017
MAPKAPK2		Knockdown reduces Liver Stage infection	Arang et al., 2017
MARK2		Knockdown reduces Liver Stage infection	Prudencio et al., 2008; Arang et al., 2017
MARK4		Knockdown reduces Liver Stage infection	Arang et al., 2017
MET		Knockdown reduces Liver Stage infection	Prudencio et al., 2008; Arang et al., 2017
PKCζ		Knockdown reduces Liver Stage infection	Prudencio et al., 2008; Arang et al., 2017
PRKWNK1		Knockdown reduces Liver Stage infection	Prudencio et al., 2008
SGK2		Knockdown reduces Liver Stage infection	Prudencio et al., 2008
STK35		Knockdown reduces Liver Stage infection	Prudencio et al., 2008
TGFBR1		Knockdown reduces Liver Stage infection	Arang et al., 2017
TYRO3		Knockdown reduces Liver Stage infection	Arang et al., 2017
ULK1		Knockdown reduces Liver Stage infection	Arang et al., 2017
WEE1		Knockdown reduces Liver Stage infection	Arang et al., 2017

A collection of host factors have also been described to impact parasite infection (Table 2B). Scavenger Receptor B1 (SRB1) and the tetraspanin CD81 both play roles in cholesterol-rich microdomain formation and are critical for hepatocyte invasion (Silvie et al., 2003; Rodrigues et al., 2008; Yalaoui et al., 2008a; Valacchi et al., 2011). More recently, it has been described that CD81 and SRB1 are involved in invasion in different species; CD81 is required for *P. yoelii* and *P. falciparum* infection, but appears to be dispensable for *P. berghei* and *P. vivax* infection. SRB1 plays a more substantial role in *P. vivax* and *P. berghei* infections (Silvie et al., 2003; Manzoni et al., 2017). It remains unknown if either protein makes contact with the sporozoite, although it has been suggested that SRB1 might directly engage the parasite, whereas CD81 indirectly impacts infection (Yalaoui et al., 2008b; Manzoni et al., 2017). The receptor tyrosine kinase EphA2 is also critical for hepatocyte infection, at least in part by engaging the parasite protein P36 (Kaushansky et al., 2015). While each of these factors contributes to the infection process, how they work in concert, and how changes in one invasion factor impacts another remain unknown. New approaches that integrate biochemical information and omics datasets are well-suited to merge with existing candidate-based research to create a more comprehensive view of the molecular components required for hepatocyte invasion (AlQuraishi et al., 2014; Gujral et al., 2014). The capacity to integrate biochemical data into a more global framework also paves the way for the identification of molecules or networks that could be targeted for intervention.

### Liver stage development

Once the parasite has taken up residence in the hepatocyte, the sporozoite dedifferentiates over the course of 12 h in rodents, or 2–3 days in humans. This process results in a rounded trophozoite, which is characterized by dramatic changes in the parasite including the disassembly of molecular and cellular structures and the expulsion of invasion machinery (Bano et al., 2007) (reviewed in Kaushansky and Kappe, 2015b; Vaughan and Kappe, 2017). Following dedifferentiation, schizogony begins, which involves the massive replication the genome, and takes place over the course of 2–10 days, depending on the *Plasmodium* species. During this time, cellular structures including lysosomes and late endosomes sequester around the parasitophorous vacuole membrane and associate with the tubovesicular network (Lopes da Silva et al., 2012; Grutzke et al., 2014). The unfolded protein response is triggered, which promotes endoplasmic reticulum stress and the survival of the Liver Stage parasite (Inacio et al., 2015; Kaushansky and Kappe, 2015a). The most dramatic change, however, is the replication of the liver stage schizont, which produces tens of thousands of merozoites (membrane-bound, haploid, red blood cell invasive forms) that eventually invade erythrocytes during blood stage development. During this process, some parasites survive, and re-wire their host cells to resist certain types of apoptotic stimuli, while others succumb to host cell apoptosis or alternative cell death stimuli (Leiriao et al., 2005; van de Sand et al., 2005; Kaushansky et al., 2013a,b; Douglass et al., 2015). While these dramatic cellular changes have been qualitatively observed, they are rarely monitored quantitatively.

Some molecular determinants have been linked to Liver Stage survival and development. For example, *Plasmodium* proteins Upregulated in Infectious Sporozoites (UIS) UIS3 and UIS4 have been hypothesized to play an active role in host nutrient acquisition, in part because of the demonstration that UIS3 associates with the host Liver Fatty Acid Binding Protein (L-FABP) (Mikolajczak et al., 2007; Blume et al., 2011; Slavic et al., 2011; Favretto et al., 2013) and localizes both proteins to the PVM (Mueller et al., 2005). Fatty acids of both host and parasite origin, including host phosphatidylcholine, have been demonstrated to be required for optimal liver stage development (Mazumdar et al., 2006; Vaughan et al., 2009; Itoe et al., 2014). How each of these components specifically contributes to the observed cellular changes, and how each factor co-opts host defenses remains unknown. A more quantitative assessment of the cellular changes that occur, matched to molecular information, will enable the development of models that describe networks of host-parasite interactions required for development of the liver stage parasite.

### Liver stage exit

Intracellular pathogens must exit their host cell in order to propagate and survive. The precise strategies they use directly impact their ability to disseminate within a host, transmit to new hosts, and engage or avoid host immune responses. Despite these important functions of exit, detailed investigations into the mechanisms governing *Plasmodium* exit have been lacking. This process is important not only for our basic understanding of liver stage development, but also for immunity. This is illustrated by the finding that the most potent stimulus of the immune system is elicited by parasites that develop through the liver stage and exit, but cannot undergo replication within the blood stage (Bijker et al., 2013).

Egress from hepatocytes occurs through the rupture of the PVM, followed by destabilization of the actin cytoskeleton, to allow the budding of merozoites from the host cell through the formation of merozoite-filled vesicles (merosomes). These structures are surrounded by a membrane of host origin (Graewe et al., 2011), and have been hypothesized to shuttle merozoites into the bloodstream to begin blood stage infection (Burda et al., 2017). This process inhibits exposure of phosphatidylserine (PS) on the outer surface of the cell, thereby simultaneously ensuring migration of parasites to the bloodstream and protection from host immune responses (Sturm et al., 2006; Tarun et al., 2006; Baer et al., 2007). Recent methodological advances have developed a platform for quantifying exit events (Stanway et al., 2009). This quantification, and a global assessment of molecular changes that occur during exit, will drive the development of models that describe networks of host-parasite interactions that underlie the exit process. Importantly, these networks could then be used to predict host and parasite determinants of dissemination to the blood stream, and the ability to engage or avoid host immune responses.

## Conclusions and new directions

Parasites must successfully navigate a wide variety of different environmental milieus, and each alternative setting presents challenges for the parasite, as well as opportunities for intervention. Here, we have described how the tools and approaches of systems biology can be deployed to more comprehensively characterize the complex interaction between parasite and host. This will inform our understanding of how the parasite and the host interact, and also facilitate future strategies to combat the parasite. Interventions that have been designed and employed without a comprehensive understanding of the complex dynamic between the *Plasmodium* parasite and its host have only partially controlled malaria in the field.

Despite the challenges, many influential leaders have called for malaria eradication in recent years (Gates, 2007; WHO, 2017). This goal is most likely to be realized if control strategies are deployed rationally with the capacity to predict how a given treatment will impact systematic changes in the parasite and host alike, to facilitate readiness for these changes. The integration of systems biology could evaluate the capacity of the parasite to circumvent new interventions, and in doing so, contribute to the success of eradication efforts. While references to the principles of systems biology first occurred decades ago, the field was established in earnest ~15 years ago with the completion of the human genome. Since then, most systems biology studies have steered clear of the complexity that is introduced when multiple genomes collide, as is the case during infection. Pathogens and their host cells have coevolved, introducing alterations to both genomes along the way (Miller et al., 1976; Zimmerman et al., 2013). What has resulted is the capacity of a pathogen to fundamentally alter the biology of its host, by changing the size, shape, composition and function of the cell. Intracellular pathogens thus are expert cell biologists, controlling the host cell to their own advantage. As such, the study of host-pathogen interactions presents an unmatched opportunity for the field of systems biology, just as the approach of systems biology presents an unmatched opportunity for the eradication of malaria.

## Author contributions

MZ, LSA, SAD, JDA, and AK were involved in the conception of the article. MZ, JDA, and AK wrote the article with assistance from LSA and SAD.

### Conflict of interest statement

The authors declare that the research was conducted in the absence of any commercial or financial relationships that could be construed as a potential conflict of interest.
